# My avatar moves with me, so I am the one acting: Avatar responsiveness supports implicit sense of agency in virtual reality

**DOI:** 10.1371/journal.pone.0351839

**Published:** 2026-06-22

**Authors:** Marika Mariano, Giulia Stanco, Caterina Negrone, Niccolò Raffa, Massimo Montanaro, Emanuele Sapio, Nadia Bolognini, Alessandro Gabbiadini, Laura Zapparoli

**Affiliations:** 1 Psychology Department and NeuroMi – Milan Centre for Neuroscience, University of Milano-Bicocca, Milan, Italy; 2 Mibtec – Mind and Behavior Technological Center, University of Milano-Bicocca, Milan, Italy; 3 IRCCS Istituto Auxologico Italiano, Milan, Italy; 4 IRCCS Orthopedic Institute Galeazzi, Milan, Italy; New York University Abu Dhabi, UNITED ARAB EMIRATES

## Abstract

Virtual reality (VR) allows users to interact with computer-generated environments in a controlled yet immersive manner. One of its most relevant features is its ability to induce virtual embodiment, the process of becoming rooted in a virtual body. A key component of this experience is the sense of agency over the action-outcomes produced in the virtual environment. However, to date, no study has jointly examined explicit and implicit senses of agency within a VR paradigm in which actions lead to consequences occurring entirely in the virtual environment. To this aim, we conducted two consecutive experiments in 70 healthy adults, who performed active or passive hand movements to switch on a virtual lightbulb. On a trial-by-trial basis, explicit agency was assessed through self-reported ratings, and implicit agency through intentional binding across three action-outcome delays (200, 400, and 600 ms). In Experiment 1 ("still avatar", n = 35), participants performed the task observing stationary virtual hands. Because this setup did not elicit the typical intentional binding phenomenon, indicating no evidence of implicit agency under these conditions, we conducted Experiment 2 in an independent sample ("responsive avatar", n = 35), in which the avatar’s hands moved synchronously with participants’ real movements. Across both experiments, participants reported significantly greater explicit agency during active than during passive movements, regardless of avatar responsiveness. In contrast, intentional binding was observed only when the avatar’s movements mirrored the participant’s real movements (Experiment 2), and exclusively for the shortest action-outcome delay (200 ms). These findings suggest that avatar responsiveness is associated with the emergence of implicit, but not explicit, sense of agency. This dissociation advances our understanding of the mechanisms underlying agency in immersive environments and informs the design of VR-based neurorehabilitation interventions that promote a stronger sense of control.

## 1. Introduction

Virtual reality (VR) allows users to interact with computer-generated environments in a highly immersive and controlled manner, offering a unique opportunity to investigate bodily self-awareness in ecological settings that go beyond traditional laboratory paradigms [1]. In particular, VR creates a three-dimensional simulated environment, allowing users to immerse themselves in and interact with realistic or imaginative scenarios that evoke genuine perceptions and reactions. One of the most relevant features of immersive VR is that it can induce virtual embodiment, namely the experience that a virtual body is experienced as one’s own body and as the locus of one’s actions [2]. This multidimensional experience is achieved through the development of a *sense of ownership* towards the virtual body and a *sense of agency* over its movements [2–5].

The *sense of ownership* refers to the continuous experience that one’s body belongs to oneself [6], and it is thought to depend on the coherent integration of multisensory signals into a stable body representation [7]. The experience of ownership has been described as comprising an implicit feeling of ownership and a more explicit judgment of ownership [8]: the first operates largely below awareness when multisensory inputs are consistent with one’s body schema, whereas the latter involves a higher-level, interpretative attribution that a body part is one’s own. In VR, both implicit and explicit ownership can be promoted by multisensory congruency, especially visuomotor correspondence between the user’s movements and the virtual body [1,2,9]. Beyond multisensory congruency, ownership is also strengthened when the avatar respects basic human-like structural features and resembles the personal characteristics [1,2].

Within the embodiment literature, the term *sense of agency* is often used to refer to the experience of controlling the movements of the virtual body [2]. However, this usage reflects a more specific, body-related aspect of agency. In the broader cognitive neuroscience literature, *sense of agency* more generally refers to the experience of being the author of one’s actions, as well as the consequences they produce in the external environment [10]. This distinction is important, as these two dimensions may rely on partially different mechanisms and may be differentially affected by features of the virtual environment. The present study focuses on this latter dimension, namely the sense of agency over action-outcomes occurring in VR.

Like ownership experience, the sense of agency should not be considered a unitary phenomenon. Rather, it is often described as a multifaceted experience comprising a non-conceptual feeling of agency and a conceptual judgment of agency [11]. The feeling of agency refers to the implicit and pre-reflective experience of agency. It is primarily grounded in sensorimotor, proprioceptive, and exteroceptive cues, as well as in the comparison between predicted and actual sensory consequences. When prediction and outcome match, actions feel fluent and self-generated; when they mismatch, actions can feel odd or not fully self-produced. By contrast, the judgment of agency reflects a higher-level, explicit and metacognitive attribution of authorship. It relies more strongly on beliefs and causal inferences, integrating additional sources of information such as intentions, thoughts, contextual cues and social knowledge [11]. Therefore, when characterising agency, it is crucial to consider that the overall experience emerges from the combination of implicit and explicit components, which may contribute differently depending on task demands and contextual ambiguity.

According to the “Cue Integration Theory”, the sense of agency arises from integrating low-level cues (sensorimotor proprioceptive and exteroceptive cues) and high-level cues (cognitive cues). The relative weight assigned to these different cues varies based on the agency dimension: the feeling of agency primarily relies on sensorimotor signals, whereas cognitive cues, such as prior beliefs, play a more prominent role in forming the judgment of agency [8,12]. This theory reconciles two previous and conflicting models: the “Comparator Model,” which emphasizes the importance of sensorimotor proprioceptive and exteroceptive signals [13], and the “Apparent Mental Causation theory”, which underscores the role of higher-level cues, such as prior experiences and beliefs [14,15].

Based on this “Cue Integration Theory”, different measures should be employed to appreciate the complexity of the agency experience fully. Explicit agency is typically assessed through direct questions that prompt participants to reflect on their actions and intentions [see, for example, 16]. In contrast, the feeling of agency is measured using indirect indices, such as the intentional binding phenomenon [17]. This phenomenon relies on the perceived compression of the temporal interval between a voluntary action and its external sensory consequences [i.e., temporal compression, see 18–20]. The validity of this phenomenon as an index of an implicit sense of agency relies on several key observations: (1) the intentional binding effect is typically observed when comparing active and passive movements, with the time between actions and consequences being systematically compressed to a greater extent for active movements; (2) this effect appears within specific time windows and only for active movements, tuned to real-life experiences [see, for example, 20,21,22]; (3) this effect is typically perturbed in patients characterised by abnormal sense of agency, such as schizophrenia patients with delusion of control [18,23] or with motor disorders [22,24].

While these theoretical frameworks have been extensively investigated in controlled laboratory settings, their application to immersive virtual environments remains only partially understood. Understanding how different dimensions of agency emerge in VR is primarily a theoretical challenge, but it is also relevant for applied contexts. Immersive VR has increasingly been used as a tool to deliver and enhance rehabilitation interventions in several neurological conditions [see 3,25], including Parkinson’s disease [26], stroke [27,28], phantom limb pain [29,30], and cerebral palsy [31,32]. In this respect, clarifying which features of the virtual experience support embodiment and a robust sense of control may provide useful theoretical foundations for the design of future VR-based interventions.

We have recently demonstrated that the sense of agency might be experienced similarly at the explicit and implicit level across different spatial contexts [33]. In particular, we modulated the ecological validity of the experimental setup to explore whether and how different spatial contexts may affect the agency experience. Participants were tested in a computer-based setting, with limited spatial information available (i.e., depth and perspective), or in a real space setting, in which participants had full access to spatial information. Results showed that participants reported a similar explicit and implicit sense of agency in both scenarios, suggesting that spatial information, regardless of whether it is more ecological or illusory, equally influences predictions about our actions and their consequences in the external, physical, or computer-based environment. Thus, it seems logical to test whether this effect may be replicated when spatial cues are presented in a virtual environment.

Several studies have investigated whether individuals may experience a sense of agency towards their voluntary actions and their outcomes in VR. Most of these studies use explicit judgments and self-report questionnaires. Participants are typically asked to execute movements with their upper limbs [34–36] or other body districts [37–41] while observing the same movement executed by their avatar in an immersive VR environment. By introducing spatial or temporal discontinuity, these experiments manipulate the visuomotor congruency between real and their avatar’s virtual movements. Participants are then asked to judge how much they felt they controlled or caused their avatar’s movements. Results show that explicit agency can be experienced in VR, as long as a visual congruency between real and virtual movements is guaranteed [see for example, 34,40,42]. Nevertheless, most of these studies did not consider the implicit dimension of agency. This is surprising, as our everyday experience of agency is usually implicit, and being asked to explicitly reason about our actions may be perceived as unnatural.

The few studies focusing on the implicit dimension of the feeling of agency, have taken advantage of the intentional binding phenomenon [see, for example, 43–46]. These studies typically require participants to estimate the perceived time interval occurring between their voluntary actions and an auditory tone. These estimates are then compared with the same measures collected in control conditions, where participants estimated the time occurring between two tones [44,47–49], or between a passive action and an auditory outcome [43,45,50,51]. Results consistently show a significant intentional binding phenomenon in VR, as typically observed in computer-based and physical settings [see, for example, 43–46].

Nevertheless, these studies leave several aspects unexplored. First, they have considered only auditory or tactile action-outcomes. It follows that the consequences of participants’ actions are not fully virtual but occur in the physical environment. In other words, participants make evaluations about an action-outcome not appearing within the virtual environment. This could affect how participants perceive the connection between actions and outcomes and the temporal delay between them. Actually, in line with Cue Integration Theory [8], the experience of implicit agency may be preserved when sensorimotor cues are strong and reliable, overriding other sources of information. Conversely, when sensory cues are unavailable or imprecise, the implicit experience of agency may be impaired, an issue that may arise when action outcomes are presented in a virtual environment [52]. To the best of our knowledge, this issue has been directly addressed only by Kong et al. (2023), who used a temporal judgment task in a VR setting. In their study, participants were placed in a virtual hallway and presented with a switched-off lightbulb positioned at either a near or far distance [53]. Through either an active or passive movement, participants triggered the lightbulb to switch on after a variable delay and were then asked to estimate the temporal interval between their action and the corresponding outcome. Their results failed to demonstrate the intentional binding effect at any distance, leading the authors to question its presence in VR. However, their study did not account for potential confounding factors, such as access to sensorimotor information in virtual reality, which may have influenced participants’ implicit sense of agency.

Second, only a limited number of studies have compared explicit and implicit agency within the same experimental paradigm, and none of them have collected these measures on a trial-by-trial basis. Studies that have assessed both implicit and explicit agency generally did so by either (a) asking participants to express their experience of control over their virtual actions and the resulting auditory outcome after a series of trials [45,46,54] or (b) using post-experimental questionnaires with broader and more generic questions about the sense of responsibility and control [44,47–49]. Measuring implicit and explicit agency on a trial-by-trial basis, along with an experimental paradigm in which the consequences of actions occur entirely in the virtual setting, may provide a more comprehensive understanding of how these components interact and influence each other within VR environments. To address these unexplored issues, we have adapted a validated experimental paradigm to measure explicit and implicit agency toward a simple action leading to a sensory outcome appearing in the VR setting.

In particular, the present study comprised two experiments (“still avatar” and “responsive avatar”) aimed at testing whether a sense of agency can be experienced in VR when sensory consequences occur in the virtual environment and whether this experience may vary according to the agency dimension (implicit vs. explicit), adapting the intentional binding paradigm in VR [21,22,33,55,56].

The two experiments differed in the degree of avatar responsiveness, namely, the extent to which the avatar’s movements were contingent upon and matched the participant’s real movements. According to established frameworks of virtual embodiment [2], such visuomotor correspondence is a key determinant of body-related sense of agency over the avatar’s movements. Thus, in the present study, avatar responsiveness can be interpreted as a manipulation of embodiment-related agency, which may in turn influence the sense of agency over action-outcomes occurring in the virtual environment.

Based on the aforementioned cognitive models on the sense of agency and the available literature on the sense of agency in VR, we can draw different predictions:

*Scenario A*. Participants may experience a sense of agency at both explicit and implicit levels. This would indicate that spatial and sensory information, even when presented virtually, enables them to make accurate predictions about their actions and sensory consequences in the virtual environment (which are crucial for the implicit sense of agency). Additionally, it allows participants to explicitly perceive themselves as the authors of their actions.
*Scenario B.* Participants may experience a sense of agency only at the explicit level, suggesting that virtual spatial and sensory information does not permit the creation of accurate predictions about our actions and their consequences in the virtual environment, leading to a lack of implicit agency. However, this information still enables participants to explicitly perceive themselves as the authors of their actions.

As the reader will see, the first experiment (“still avatar”) yielded results coherent with Scenario B. However, we believe that some features of the experimental setting may have influenced the results, prompting us to conduct a second experiment (“responsive avatar”) using the same task but fostering virtual embodiment. Indeed, at the very beginning of the first experiment (“still avatar”), participants saw their avatars in a congruent position but remained still in the virtual environment (even if, in this phase of the experiment, subjects were instructed to stay still, thus, no incongruence between the participants and the avatar was detectable). However, we believe that this lack of correspondence between real and avatar movements may have affected our subsequent results.

In the second experiment (“responsive avatar”), the avatars moved consistently with the participants’ actual hand movements. This modification aimed to enhance virtual embodiment by providing more realistic visuomotor feedback, thereby offering a clearer understanding of the agency experience in VR.

## 2. Experiment 1 – “Still avatar”

### 2.1 Materials and methods

#### Participants.

An a priori power analysis was conducted using Jamovi (*pamlj* module [57]). The analysis targeted the Action * Delay interaction, characterised by 2 degrees of freedom, based on an effect size of ηp² = .20 observed in a previous study using the same experimental paradigm [21]. Assuming a repeated-measures factorial design and a significance level of α = .01, the analysis indicated that a sample size of 32 participants would provide 80% statistical power to detect an effect of this magnitude.

To consider possible dropouts and outliers, we recruited thirty-five adult participants (mean age, 22.5 ± 2.7 years; mean education, 14.4 ± 3.11 years; male/female ratio, 12:23) with no history of neurological or psychiatric illness. One participant was excluded because it was discovered after data collection that they were taking antidepressant medication at the time of the experiment.

Participants were university students recruited via word of mouth and through official university channels. Recruitment took place between November 26, 2022, and April 17, 2024. The study protocol was conducted in accordance with the ethical standards of the Declaration of Helsinki and was approved by the local Ethics Committee (Committee for Research Evaluation, Department of Psychology, University of Milano-Bicocca; protocol number RM-2022–510). All participants provided written informed consent prior to taking part in the experiment.

#### Experimental setting and procedure.

**Fig 1:**
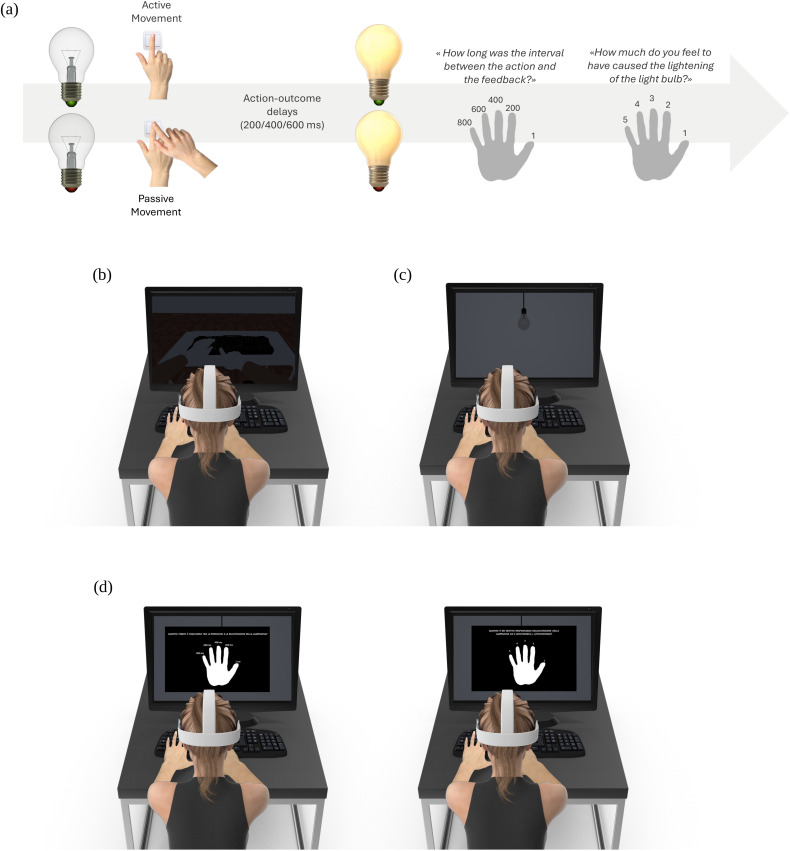
Graphical illustration of the experimental “lightbulb” paradigm and settings. **(a)** Trial timeline (for both active and passive conditions): participants are presented in a VR scenario with a switched-off lightbulb with a green or red base. The green base indicated participants to press a button with their right finger at their own time to switch on the lightbulb (active condition), and the red base invited participants to stay still and wait for the experimenter to press their finger on the button (passive condition). In both conditions, the button press caused an action consequence: the lightbulb switched on after a variable delay (200, 400, or 600 ms) from the active or passive movement. Then, participants were asked to rate the perceived time interval between the button press and the lightening of the lightbulb (to measure the intentional binding phenomenon, i.e., implicit measure of agency) and to judge how much they felt to have produced, with their action, the lighting on of the lightbulb (judgment of agency, i.e., explicit measure of agency). **(b)** Participants observed their virtual hands stationary on a table before the experiment, with the instructions to stay still. **(c)** VR scenario presented during the experimental session: an avatar sitting, with its hands still on a keyboard located on the table and a turned-off lightbulb in the centre of the empty room; on the table, also, a keypad was visible, congruently with the real environment. Please note that participants did not look at their hands during the experimental trials. **(d)** Participants saw the possible options on the floating panel in HMD and made their judgments with a keypad on explicit (on the left) and implicit (on the right) agency.

We measured the sense of agency experienced while executing a daily life action, namely, switching on a lightbulb (Fig 1a).

Before the experimental session, participants underwent for five minutes a familiarisation phase. They were seated comfortably on a chair in front of a table, wearing a Meta Quest 2 headset. In this virtual scenario, developed using the Unity game engine modelling environment (ver. 2022.3.17f1) and C# scripting, they found themselves in an empty room; a gender-matched virtual body was sitting on a chair, with its virtual hands resting on the table (Fig 1b). Participants were invited to position their hands in alignment with the virtual avatar’s hands and to remain still until the experiment began. Accordingly, the virtual avatar remained stationary.

Participants were then presented with a new virtual scenario in which the avatar sat in front of a table with a turned-off lightbulb in the centre of the empty room. They were instructed to observe the environment, which was spatially congruent with the real-world setting (Fig 1c).

To modulate the action-outcome causality in the experience of agency, we varied both the intentionality of movement (active vs. passive button press) and the time interval between the action and its outcome. Specifically, the lightbulb had either a red or green base (Fig 1a). In the active condition, the lightbulb base was green, and participants were instructed to press a button located both physically and virtually on the table in front of them using their right index finger. In the passive condition, the lightbulb base was red, and participants were instructed to remain still while an experimenter pressed their right index finger to produce a passive action. After either an active or passive button press, the lightbulb switched on following a variable delay of 200, 400, or 600 milliseconds. These temporal delays were introduced to investigate their impact on the perceived agency over the action-outcome (temporal) relationship [see 58].

Throughout the experiment, the keyboard was visible in the VR scene in a physically congruent position relative to the participant (Fig 1b). During the main task, however, the avatar’s hands were static, and no visually congruent button-press movements were displayed. Moreover, participants were instructed to maintain their attention on the lightbulb (i.e., the action-outcome), which was not simultaneously visible with the hand and keyboard. Compliance was monitored online by the experimenter via a mirrored display of the VR scene.

After the lighting on of the lightbulb, participants rated the perceived time interval between their button press and the lightbulb lighting up (to measure the intentional binding phenomenon, implicit agency dimension). Participants chose from five response options: 1, 200, 400, 600, and 800 milliseconds. The lowest (1 milliseconds) and the highest (800 milliseconds) answer options were included to allow underestimation and overestimation of the actual temporal interval.

Participants were also asked to judge the perceived level of agency, reporting how much they felt they had produced, with their action, the lightening of the lightbulb (i.e., explicit agency dimension). Again, they had to select one of five options on a visual scale, from 1 (not at all caused by me) to 5 (entirely caused by me).

Participants could see the options on a floating panel for each evaluation directly in the VR simulation, while the estimations were reported using a keypad on the table in front of them (Fig 1d), which was also congruently represented in the VR environment.

We administered 72 trials, equally distributed between active and passive trials (36 for each condition), with 12 trials for each of the three action-outcome delays.

#### Statistical analyses – Explicit agency.

Data were analysed using R (version 4.4.2; R Core Team, 2024) with a within-subject (repeated-measures) 2 × 3 design. For the explicit experience of agency, the dependent variable was the agency rating provided by participants in response to the question: “How much do you feel you caused the lightbulb to light up?” responses were given on a Likert scale ranging from 1 (not at all caused by me) to 5 (entirely caused by me). Agency ratings were then averaged for each participant, separately for each combination of action type (active vs. passive) and temporal delay, resulting in a single mean value per condition. These represented the dependent variable of the model, while the factors “Action” (active/passive) and “Delay” (200/400/600 ms) were the independent variables. The outlier exclusion criterion was defined prior to data analyses, and it was performed at the participant level using condition-specific mean values (i.e., each participant’s average score within each Action × Delay condition). Participants whose mean value exceeded 1.5 times the interquartile range [59] in at least one condition were excluded from that analysis.

Based on this, four participants were classified as outliers (total sample size for this analysis: 30 participants). After removing these outliers, given the nonparametric distribution of the data (trough visual inspection of the Q-Q plot, 57), we performed a non-parametric Aligned Rank Transform [ART, 60] ANOVA using the ARTool package in R. Significant effects were explored with post-hoc tests corrected for multiple comparisons.

#### Statistical analyses – Implicit agency.

We first calculated the variable called “time compression”, namely the difference between the actual and estimated duration of the action outcome delay. This measure was taken as an indirect measure of the sense of agency (the greater the compression, the higher the implicit sense of agency), in line with the intentional binding phenomenon [17]. Negative time compression values indicate a stronger time compression, and a stronger time compression in active compared to passive conditions indicates a significant intentional binding effect (and thus enhanced implicit sense of agency).

We considered these values, averaged for each participant, for every combination of action type and temporal delay, as the dependent variable of the model, while the factors “Action” (active/passive) and “Delay” (200/400/600 ms) were the independent variables, according to a within-subject (repeated-measures) 2 × 3 design. Based on the outlier detection procedure described in the previous paragraph, none of the participants was classified as outlier (total sample size for this analysis: 34 participants).

Given the parametric distribution of the data [trough visual inspection of the Q-Q plot, 57], we tested this statistical model using a parametric repeated-measures ANOVA. Significant effects were explored through planned post-hoc comparisons, corrected for multiple comparisons using the Bonferroni method.

#### Statistical analyses – Exploratory correlation analyses between implicit and explicit sense of agency measures.

To examine the relationship between explicit and implicit measures of agency, we averaged agency ratings (explicit agency) and correlated them with time compression values (implicit agency) across different temporal delays for the active conditions. Significant p-values were corrected using Bonferroni adjustment for the planned comparisons.

### 2.2 Results

#### Explicit agency.

**Fig 2:**
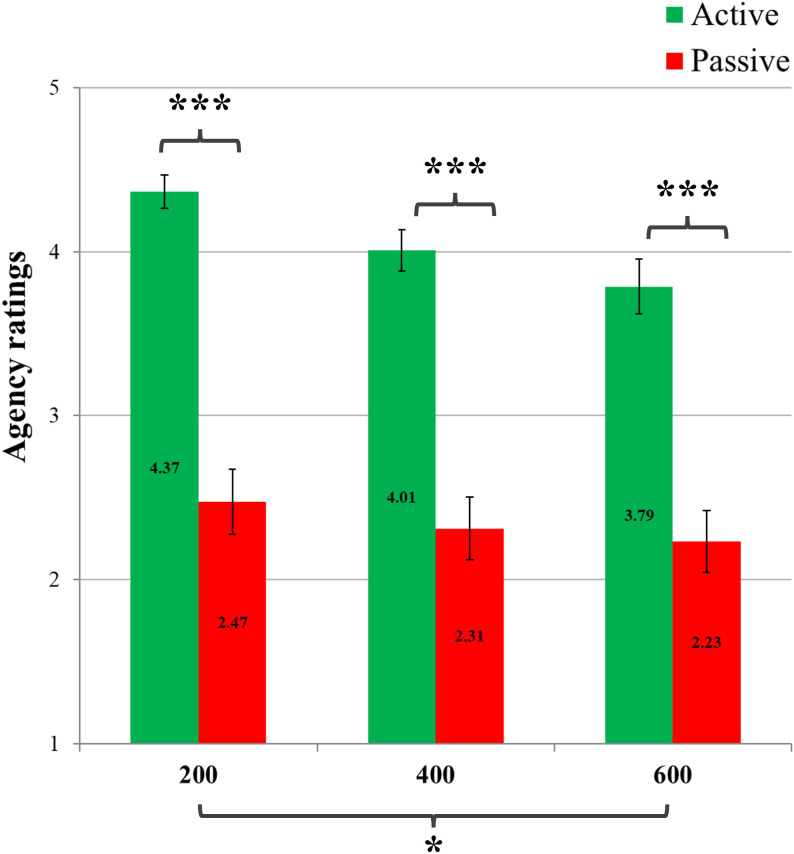
Mean and standard error values of agency judgments. Participants show greater explicit agency for active actions than passive ones. *** p_corrected_ < .001, ** p_corrected_ < 0.01, * p_corrected_ < 0.05.

The ART ANOVA revealed a significant main effect of Action (F(1,145) = 181.81, p < 0.001), indicating that ratings were higher in Active compared to Passive conditions, and Delay (F(2,145) = 3.17, p = 0.045). The Action*Delay interaction (F(2,145) = 0.37, p = 0.69) was not significant. Tukey-corrected post-hoc pairwise comparisons revealed that agency ratings were higher for the 200ms compared to the 600ms action-outcome delay (200–400 ms: t(145) = 1.63, p corrected = 0.24; 200–600 ms: t(145) = 2.48, p corrected = 0.04; 400–600 ms: t(145) = 0.85, p corrected = 0.67, see Fig 2).

#### Implicit agency.

We found a main effect of the factor Action (F(1,33)=11.29, p = 0.002, η^2^p=0.25) and Delay (F(2,66)=47.91, p < 0.001, η^2^p=0.59) and a significant Action*Delay interaction (F(2,66)=10.58, p < 0.001, η^2^p=0.24). We explored the Action*Delay interaction with Bonferroni corrected planned post-hoc comparisons.

**Fig 3:**
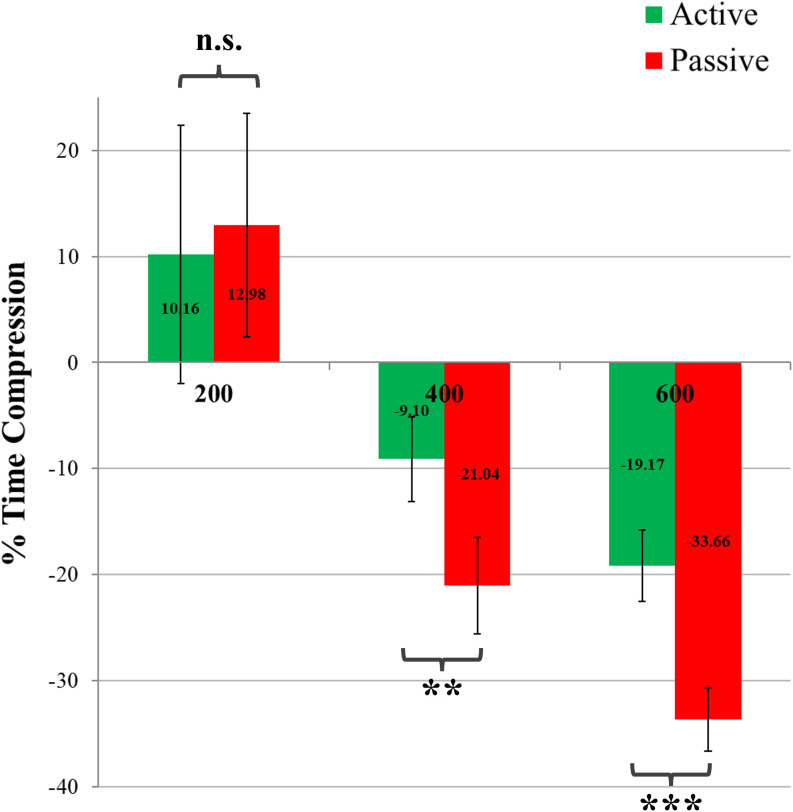
Mean and standard error of time compression values. Participants do not exhibit intentional binding in any of the temporal delays. Time compression values are reported as a percentage of the temporal delay considered. *** p_corrected_ < .001, ** p_corrected_ < 0.01, * p_corrected_ < 0.05.

We observed greater time compression for passive actions than active ones at 400ms and 600ms, thus showing repulsion rather than implicit sense of agency for the longer action-outcome delays (200ms: t(33)=0.38, p corrected >0.99, 400ms: t(33)=−3.14, p corrected = 0.012, 600ms: t(33)=−4.10, p corrected<0.001, see Fig 3).

In other words, we could not replicate the intentional binding phenomenon, thus indicating that our participants did not experience an implicit sense of agency.

#### Exploratory correlation analyses between implicit and explicit sense of agency measures.

The correlation between the judgments of agency and the time compression values was not significant for all the temporal delays and type of actions investigated (Passive action: 200ms: Spearman’s rho (28)=−0.04, p = 0.82; 400ms: Spearman’s rho (28)=−0.13, p = 0.51; 600ms: Spearman’s rho (28)=0.07, p = 0.70; Active action: 200ms: Spearman’s rho (28)=−0.30, p = 0.10; 400ms: Spearman’s rho (28)=−0.14, p = 0.45; 600ms: Spearman’s rho (28)=−0.27, p = 0.16).

### 2.3 Interim discussion

In this first experiment (“still avatar”), we assessed explicit and implicit sense of agency on a trial-by-trial basis while participants performed daily life actions within a VR setting.

At the explicit level, we observed higher agency ratings in active compared to passive conditions across all temporal delays, suggesting that individuals can experience agency for their virtual actions and following outcomes, similar to what was found in real space and computer-based settings [33].

Conversely, we could not replicate a significant intentional binding effect: time compression values were similar in active and passive actions in the shortest temporal delay, and stronger for passive conditions in the longer temporal delays. This suggests that our participants did not experience implicit agency for actions whose consequences are not produced in the virtual environment.

These results are consistent with the hypothesised Scenario B indicating that participants experience explicit, but not implicit, agency when performing actions in a virtual spatial contest with “virtual” sensory consequences. However, some features of the experimental setting may have influenced these results. Notably, in this experiment, the avatar did not provide congruent visuomotor feedback during the actions: although the participants saw their avatars in a congruent position, it remained still in the virtual environment. Importantly, participants were instructed to keep their attention on the lightbulb and therefore did not continuously monitor their hands during action execution. Nevertheless, the absence of coherent visuomotor evidence (and the resulting mismatch between participants’ movements and the visual state of the avatar) may have weakened the sensorimotor predictions that contribute to the implicit sense of agency, thereby contributing to the absence of intentional binding in this experiment.

Therefore, we designed a second experiment (“responsive avatar”) to investigate whether tracking the avatar’s movements may influence the implicit experience of agency thanks to an enhanced virtual embodiment driven by a more realistic visuomotor feedback.

## 3. Experiment 2 – “Responsive avatar”

With this experiment, we wanted to (i) evaluate whether tracking the avatar’s movement can restore the implicit experience of agency and (ii) assess the relationship between agency measures and the explicit experience of ownership.

In this second experiment (“responsive avatar”), conducted on an independent sample of participants, we maintained the same experimental design as in Experiment 1 (“still avatar”), modifying the VR experience by allowing the avatar to match participants’ movements. The participant’s avatar was designed by using an online generation tool (i.e., ReadyPlayerMe). However, avatars generated with this tool do not allow an advanced hand-tracking mechanism. Consequently, the generated model was edited through a *rigging* procedure. Rigging in VR Unity involves creating a skeletal structure for 3D models, which allows for realistic movements and animations in a VR scene. This includes setting up bones, joints, and constraints to allow natural and fluid movements. In particular, the avatar’s arms, hands, and fingers were adapted using this modelling technique. To animate the avatar based on the user’s actions, we made a dedicated C# script. This script is responsible for tracking the user’s head, arms, hands, and fingers movements and updating the avatar’s position accordingly. Indeed, matching participant’s arms, hands, and fingers movements was essential for the goals of the present work. All participants experienced the virtual scenarios through a Meta Quest 2 head-mounted display.

Moreover, to facilitate the process of embodiment, a virtual mirror was introduced in the VR environment to allow users to see and control their avatar’s movements in real-time, thus allowing immediate visual feedback that fosters a stronger connection between the user and the virtual body [61].

Moreover, in this second experiment, the explicit experience of ownership over the virtual limb was also assessed [44,46].

### 3.1 Materials and methods

#### Participants.

We recruited a new and independent sample of thirty-five adult participants (mean age, 22.8 ± 2.92 years; mean education, 14.46 ± 1.79 years; male/female ratio, 13:22) with no neurological or psychiatric illness history. Similarly to Experiment 1 (“still avatar”), participants were university students recruited through word of mouth and the official channels provided by the university. Recruitment took place between November 26, 2022, and April 17, 2024. The study protocol was conducted in accordance with the ethical standards of the Declaration of Helsinki and was approved by the local Ethics Committee (Committee for Research Evaluation, Department of Psychology, University of Milano-Bicocca; protocol number RM-2022–510). All participants provided written informed consent prior to taking part in the experiment.

The sample size was determined based on the same power analysis described in Experiment 1 (“still avatar”).

#### Experimental setting and procedure.

**Fig 4:**
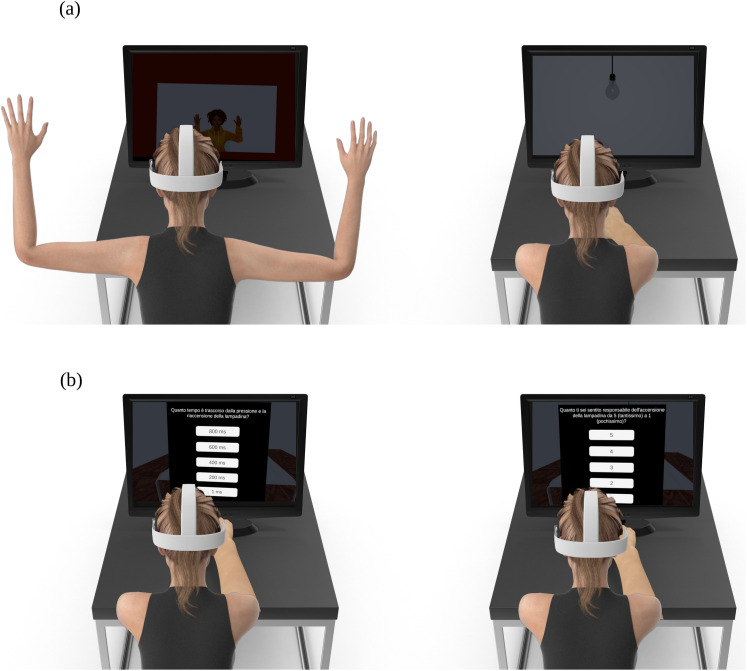
Graphical illustration of the (a) familiarization phase of Experiment 2 (“responsive avatar”) and (b) floating panel used by participants to report their evaluations.

The experimental setting and procedure were the same as in Experiment 1 (“still avatar”). However, the familiarisation phase changed: in this virtual scenario, participants found themselves in an empty room while a gender-matched virtual body was sitting on a chair in front of a mirror. Participants were invited to look at their avatar in the mirror while executing a series of instructed and free movements. Thus, unlike Experiment 1, during the five-minute familiarisation phase, participants could see their virtual hands moving consistently with their real hand movements (Fig 4a).

Following the familiarisation phase, participants started the experimental session, which was identical to Experiment 1 (“still avatar”). As in Experiment 1, participants could see a virtual button positioned in a physically congruent location with respect to the real response device. However, unlike Experiment 1, in Experiment 2 (“responsive avatar”), participants experienced the avatar’s hand moving coherently with their own movements and performed the button press in the virtual environment. In addition, participants provided their evaluations differently: they made their choice by touching, with their virtual fingers, an interactive floating panel displayed in the VR scenario, emulating a touchscreen interaction (Fig 4b). As in Experiment 1 (“still avatar”), participants were instructed to maintain their attention on the lightbulb and to avoid looking down at their hands during the main task, as the hand/button and the lightbulb could not be viewed simultaneously. Compliance with this instruction was continuously monitored by the experimenter via a mirrored display of the VR scene.

Similarly to previous studies [44,46], at the end of the experimental session, we asked participants to retrospectively rate their subjective feeling of ownership experienced over their avatar, presenting them with three questions, adapted from Rossetti et al. [62]: (Q1) “*It seemed like I was looking directly at my own hand*”, (Q2) “*It seemed like the avatar’s hand belonged to me”*, (Q3) “*It seemed like the avatar’s hand was my hand*”, (Q4) “*It seemed to feel the movement of my hand where the avatar’s finger was moving*”, on a visual analogue scale from 0 (not at all) to 100 (completely).

#### Statistical analyses – Explicit agency.

Data were analysed by using the software R (Version 4.4.2), in a within-subject (repeated-measures) 2 × 3 design. Explicit agency ratings represented the dependent variable of the model, while the factors “Action” (active/passive) and “Delay” (200/400/600 ms) were the independent variables.

Based on the outlier detection procedure described for Experiment 1, one participant was classified as outlier (total sample size for this analysis: 34 participants). Given the nonparametric distribution of the data (trough visual inspection of the Q-Q plot, [57]), we performed a non-parametric Aligned Rank Transform [ART, 60] ANOVA using the ARTool package in R. Significant effects were explored with post-hoc tests corrected for multiple comparisons.

#### Statistical analyses – Implicit agency.

Data were analysed using the software Jamovi (Version 2.3.21.0) with a within-subject (repeated-measures) 2 × 3 design. We considered time compression values as the dependent variable of the model, while the factors “Action” (active/passive) and “Delay” (200/400/600 ms) were the independent variables. Based on the outlier detection procedure described for Experiment 1, four participants were classified as outliers (total sample size for this analysis: 31 participants).

After removing these outliers, given the parametric distribution of the data [trough visual inspection of the Q-Q plot, [57], we tested this statistical model using a parametric repeated-measures ANOVA. Significant effects were explored through planned post-hoc comparisons, corrected for multiple comparisons using the Bonferroni method.

#### Statistical analyses – Exploratory correlation analyses between implicit and explicit sense of agency measures.

To examine the relationship between explicit and implicit measures of agency, we averaged agency ratings (explicit agency) and correlated them with time compression values (implicit agency) across different temporal delays.

#### Statistical analyses – Exploratory correlation analyses between sense of agency measures and sense of ownership.

First, to assess whether participants experienced a sense of ownership toward the virtual avatar, we conducted a one-sample t-test comparing ownership ratings to 0, which represented the absence of ownership on the visual analogue scales (range: 0–100). This analysis aimed to verify whether the embodiment manipulation successfully elicited a subjective feeling of ownership, even in the absence of a control condition.

Next, to assess the correlation between explicit and implicit agency and the sense of ownership, we averaged ownership ratings and correlated them with the judgments of agency (for explicit agency) and with time compression values (for implicit agency). The significant p-values were corrected with Bonferroni planned multiple comparisons.

### 3.2 Results

#### Explicit agency.

**Fig 5:**
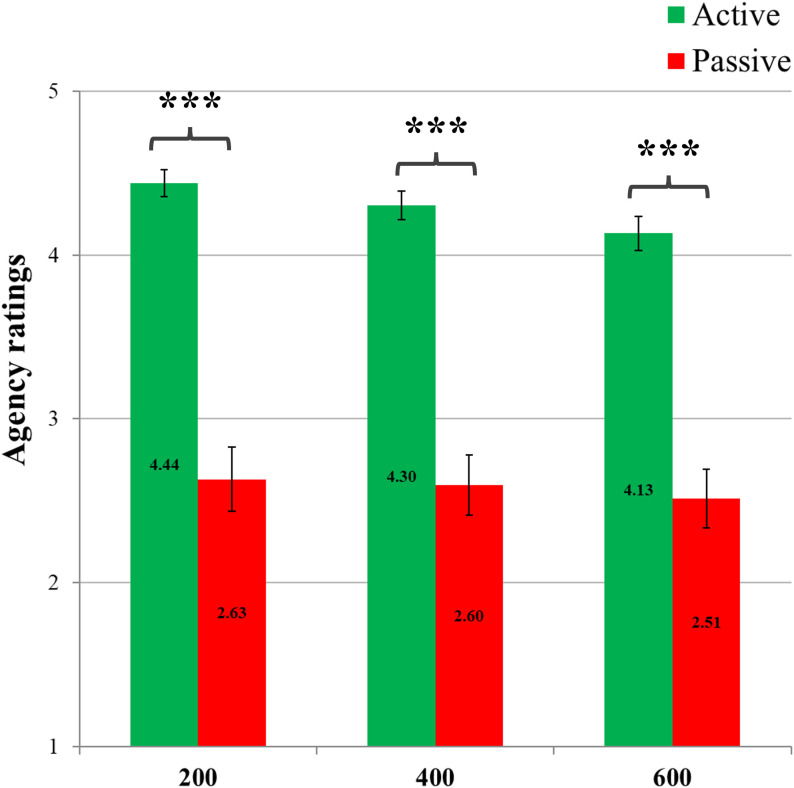
Mean and standard error values of agency judgments. Participants show greater explicit agency for active actions than passive ones. *** p_corrected_ < .001, ** p_corrected_ < 0.01, * p_corrected_ < 0.05.

The ART ANOVA revealed a significant main effect of Action, F(1,165) = 369.80, p < 0.001, indicating that ratings were higher in Active than Passive conditions. The main effect of Delay (F(2,165) = 1.77, p = 0.17) and the Action*Delay interaction (F(2,165) = 0.29, p = 0.75), were both non-significant. See Fig 5.

#### Implicit agency.

We found a main effect of the factor Delay (F(2,60)=22.52, p < 0.001, η^2^p=0.43) and a significant Action*Delay interaction (F(2,60)=17.15, p < 0.001, η^2^p=0.36). The main effect of the factor Action was not significant (F(1,30)=2.11, p = 0.16, η^2^p=0.07). We explored the Action*Delay interaction with Bonferroni corrected planned post-hoc comparisons.

**Fig 6:**
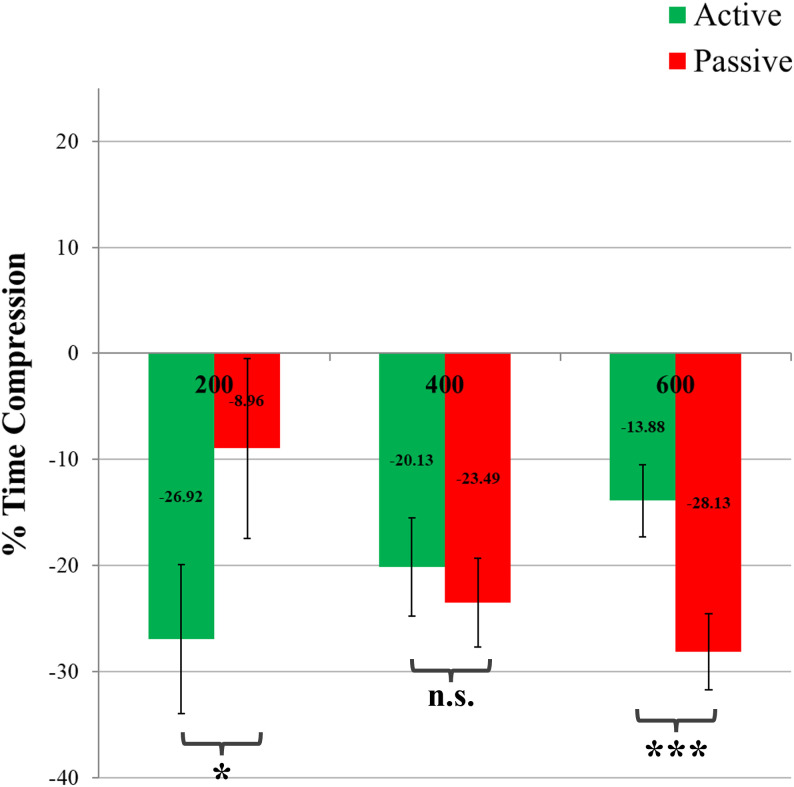
Mean and standard error of time compression values. Participants exhibit stronger intentional binding for active actions whose consequences were temporal contingent to the outcome. Time compression values are reported as a percentage of the temporal delay considered. *** p_corrected_ < .001, ** p_corrected_ < 0.01, * p_corrected_ < 0.05.

We observed a significant sense of implicit agency only at 200ms, with higher temporal compression values in active vs passive trials for the 200ms action-outcome delay (t(30)=2.82, p corrected = 0.027). Moreover, we observed greater time compression for passive actions than active ones at 600ms (t(30)=−3.88, p corrected<0.001). We did not observe a significant difference at 400ms of action-outcome delay (t(30)=−0.66, p corrected>0.99). See Fig 6.

#### Exploratory correlation analyses between implicit and explicit sense of agency measures.

**Fig 7:**
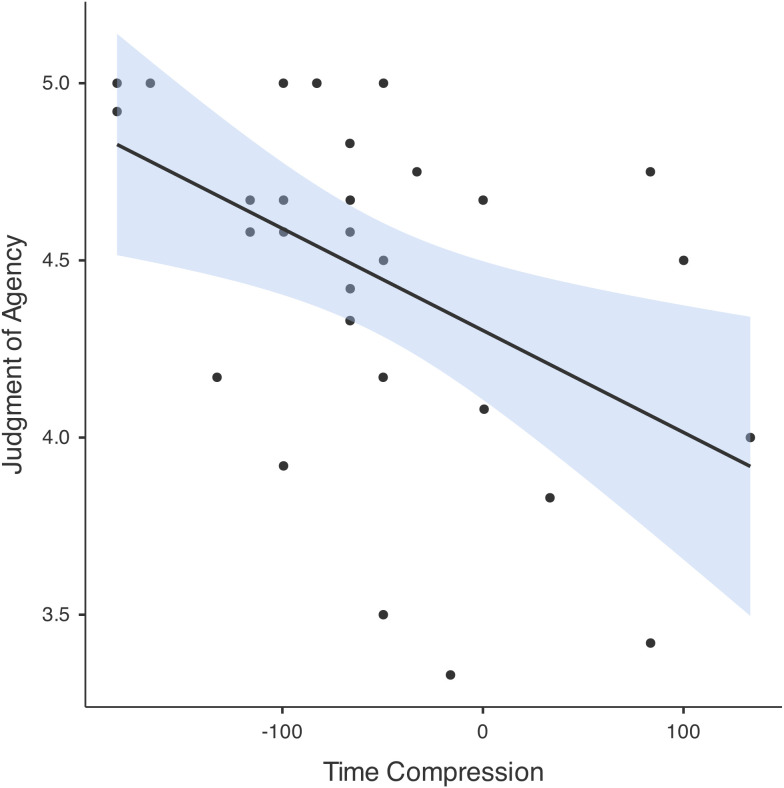
Negative correlation between time compression and judgment of agency in the active 200 ms delay condition. A significant inverse relationship was found (Spearman’s ρ = –0.48, p = 0.007, p corrected = .042), indicating that greater (i.e., more negative) time compression values were associated with higher subjective agency judgments.

Results show a significant negative correlation between the judgments of agency and the time compression values only for the shortest temporal delay in case of active actions (Fig 7, Passive action: 200ms: Spearman’s rho (29)=0.01, p = 0.97; 400ms: Spearman’s rho (29)=0.22, p = 0.23; 600ms: Spearman’s rho (29)=−0.03, p = 0.88; Active action: 200ms: Spearman’s rho (29)=−0.48, p = 0.007, p corrected = 0.042; 400ms: Spearman’s rho (29)=−0.18, p = 0.33; 600ms: Spearman’s rho (29)=0.06, p = 0.77). It is important to highlight that the negative correlation observed at the shortest action-outcome delay (200ms) reflects the fact that more negative time compression values correspond to stronger intentional binding. Therefore, this result indicates that greater implicit agency was associated with higher explicit agency ratings.

#### Exploratory correlation analyses between sense of agency measures and sense of ownership.

The Ownership ratings differed significantly from 0, indicating that participants reported moderate levels of ownership over the virtual body (M = 62.46; SD = 21.64; t(34)=17.1, p < .001).

The correlation between the judgments of agency and the sense of ownership was not significant for all the temporal delays and type of actions investigated (Passive action: 200ms: Spearman’s rho (32)=0.08, p = 0.66; 400ms: Spearman’s rho (32)=0.15, p = 0.40; 600ms: Spearman’s rho (32)=0.11, p = 0.53; Active action: 200ms: Spearman’s rho (32)=−0.21, p = 0.23; 400ms: Spearman’s rho (32)=−0.35, p = 0.042, p corrected = 0.13; 600ms: Spearman’s rho (32)=−0.31, p = 0.08).

A similar result was found for time compression values, which did not correlate with ownership ratings (Passive action: 200ms: Pearson’s r (29)=0.35, p = 0.06; 400ms: Pearson’s r (29)=0.24, p = 0.19; 600ms: Pearson’s r (29)=0.16, p = 0.40; Active action: 200ms: Pearson’s r (29)=0.31, p = 0.09; 400ms: Pearson’s r (29)=0.18, p = 0.33; 600ms: Pearson’s r (29)=0.03, p = 0.85).

### 3.3 Interim discussion

Similar to what was observed in Experiment 1 (“still avatar”), results from Experiment 2 (“responsive avatar”), on an independent sample of participants, showed higher agency ratings in active compared to passive conditions for all the temporal action-outcome delays, hence documenting the development of a sense of explicit agency in the VR setting.

Crucially, a significant experience of agency was now recorded also at the implicit level: participants showed a significant intentional binding effect, with more negative time compression values for active actions compared to passive ones, when there was a temporal contingency between their actions and consequences. Actually, implicit agency was significant only when the interval between the participants’ button presses and the lightbulb illumination was 200 ms. This finding aligns with previous studies investigating implicit agency in both computer-based and real-world settings [e.g., 21,22,33], and are in line with previous studies showing that sense of agency decreases along with the increase in delay [12,20,58].

Finally, no significant correlations were found between either implicit or explicit measures of agency and the reported sense of ownership.

Overall, these results are consistent with the hypothesised Scenario A: the possibility of observing our movements in the virtual environment through an avatar, even when not requested by the task, may be one factor associated with the emergence of the implicit experience of agency.

## 4. General discussion

Recent research has demonstrated the potentialities of immersive VR as a complementary rehabilitation tool [see 3,25] for treating different neurological disorders [see, for example, 26,28,30]. These studies underscore the importance of becoming rooted in the virtual body and having a sense of control over the avatar’s movements for the treatment to be effective. This includes the so-called sense of agency, namely the feeling of controlling our actions and the produced outcomes [10].

We conducted two independent experiments to explore the explicit and implicit dimensions of agency for actions leading to consequences appearing in VR. Both experiments employed the same experimental setting and procedure, differing in avatar responsiveness and in the modality used to collect participants’ responses. In the first experiment (“still avatar”), participants observed their avatars positioned anatomically congruent to their own bodies, but the avatars remained stationary in the virtual environment. In contrast, during the second experiment (“responsive avatar”), participants’ real movements were precisely mirrored by their avatars in the virtual environment. Importantly, during the experimental session, our participants were instructed to focus solely on the lightbulb and refrain from looking at their hands. As a result, during the task (pressing a button), they did not directly observe their virtual hands’ movements. This choice does not reflect a mere design limitation, but rather a controlled methodological trade-off motivated by the need to preserve a validated intentional-binding paradigm and to ensure accurate temporal judgments of VR-based outcomes. Actually, this choice ensured continuity with our standard implementation of the paradigm in previous non-VR studies [e.g., MRI/TMS settings; see [21,22,33,56], where participants cannot observe their hands during action execution. Furthermore, because the hand/button and the lightbulb could not be viewed simultaneously; thus, directing attention to the lightbulb was necessary to guarantee consistent visual processing of the action-outcome.

In the first experiment (“still avatar”), results show that participants experienced explicit sense of agency during active actions. This is in line with our previous findings using computerised-based scenarios in the real context, whereby participants provided higher agency judgments when they actively controlled their actions rather than being passive [33,56].

Instead, we do not observe a significant intentional binding phenomenon (i.e., similar time compression values in active and passive conditions), indicating that participants did not experience an implicit sense of agency in a VR environment. This pattern may reflect reduced access to the sensorimotor cues that typically contribute to implicit agency, which may have weakened participants’ predictions about the sensory consequences of their actions in the virtual environment. This result is consistent with findings by Kong and colleagues (2023), who employed a similar experimental task in a virtual reality setting and also failed to detect an intentional binding effect across all tested temporal delays and spatial distances [53].

To further test this hypothesis, we conducted a second experiment in which the avatar’s head, hands and fingers move coherently with the individuals’ movements (Experiment 2 - “responsive avatar”). In this case, participants experienced both an explicit and implicit sense of agency. These results are consistent with the possibility that avatar movement tracking may support the emergence of the implicit experience of agency. This finding complements the results of Wiesing and colleagues (2024), demonstrating an augmented implicit sense of agency when participants could see their avatars performing virtual actions, compared to conditions where they moved without visual feedback of their actions [43]. This may also help explaining why Kong and colleagues (2023) failed to observe an implicit sense of agency in their virtual reality setting. In their study, participants interacted with a non-ecological virtual environment, where only a lightbulb was visible in the middle of a hallway [53]. Crucially, they lacked access to sensorimotor cues typically provided by a responsive avatar, similar to the setup used in our Experiment 1 (“still avatar”), thus limiting the emergence of the implicit agency.

Importantly, participants exhibited a significant intentional binding effect, with more negative time compression values for active compared to passive actions, specifically with the shortest action-outcome delay (200 ms), during the main task. This finding is consistent with previous studies investigating implicit agency in both computer-based and real-world contexts [e.g., 21,22,33]. Importantly, about 200 ms is the latency that can be measured in real life between the time when we press a light switch and the time that a conventional lightbulb takes to be fully on [63]. Overall, these findings support prior evidence that increasing the delay between action and outcome reduces implicit experience of agency, underscoring the critical role of temporal contingency and daily life experience of a given action and its usual effect in agency attribution even in VR settings [12,20,58].

Interestingly, a counterintuitive reversed effect was consistently observed at the longest temporal delay (600 ms) in both Experiments 1 (“still avatar”) and 2 (“responsive avatar”), with stronger time compression reported for passive compared to active trials. This finding aligns with our previous studies [33,64], suggesting that the effect is not specific to action execution within a virtual environment. One possible explanation for these results is the influence of the experimenter’s physical presence on participants’ implicit sense of agency. Zapparoli et al. (2022) showed that implicit agency can extend to others’ actions, so-called vicarious agency, especially when sensorimotor information is available [65]. In our study, during passive trials, the experimenter sat beside participants and physically moved their fingers to press the button, providing full sensorimotor access. This may have led participants to generate motor predictions, enhancing their sense of agency over the outcome. The unusual 600 ms delay and the physical proximity of the experimenter might have further prompted attribution to the experimenter’s action [64]. However, we acknowledge that this interpretation remains hypothetical and warrants direct investigation in future research.

On the other hand, the explicit dimension of agency remained unaffected by the manipulation introduced in the second experiment, with agency judgments remaining significantly higher in active vs passive actions. This result seems to contradict previous findings indicating that visual movement congruency is crucial for recognizing oneself as the author of an action [see, for example, 34,40,42]. We believe this apparent contradiction may be explained by the characteristics of our experimental design. In previous studies, participants were typically asked to observe and judge their sense of agency based on their own virtual movements. In contrast, our participants focused on evaluating the consequences of their actions rather than the movements themselves, which may account for the observed differences in explicit agency judgments.

Together with the experience of agency, the sense of ownership, namely the feeling of owning a body, is a crucial component of the embodiment process. In our second experiment, characterised by more realistic visuomotor feedback, we also assessed the explicit experience of ownership. Results indicate that participants reported ownership over the virtual body. Notably, this measure did not correlate with our explicit and implicit agency measures. This result may reflect an important conceptual distinction: within embodiment frameworks, sense of agency typically refers to the sense of motor control over the virtual body itself [2], whereas in the present study, agency was operationalised as the sense of control over action-outcomes [10]. Accordingly, the lack of correlation may stem from the fact that these measures tap conceptually distinct components of self-representation in VR. However, this measure should be interpreted cautiously, as it does not include a control condition and primarily serves as a manipulation check. Future studies should examine embodiment more comprehensively by assessing, within the same experimental paradigm, the three core components of embodiment – self-location, body-related agency, and ownership [2] – thereby clarifying how different forms of agency (body-related vs. outcome-related) interact with each other in immersive virtual environments.

### Implications for cognitive models of sense of agency and the development of VR scenarios

The dissociation between the two dimensions of agency observed in our first experiment (“still avatar”) and the comparison of the two experiments also provide some important hints for discussing the validity of cognitive theories on the sense of agency described at the beginning of this paper.

These findings suggest that implicit agency in VR may be more likely to emerge under conditions that support avatar responsiveness. Notably, our findings also suggest that continuous online visual feedback of one’s own action execution may not be necessary for implicit agency to emerge in VR. During the experimental trials, participants were instructed to keep their attention on the lightbulb and therefore did not constantly monitor their hands while pressing the button. Nonetheless, in Experiment 2 (“responsive avatar”), we observed reliable intentional binding, consistent with the idea that establishing a coherent visuomotor mapping during the familiarisation phase and thus learning that one’s actions are effective within the surrounding virtual context, may be sufficient to support implicit agency for virtual consequences. This aligns with everyday action experience, where people experience a feeling of coherence in the ongoing flow of action (e.g., pressing a button to switch on a light) even without visually monitoring their hands, provided that the sensorimotor mapping is stable and the action-outcome relationship is reliable. Explicit agency, however, was preserved across both experiments, indicating that conscious agency judgments can remain robust even when visuomotor coherence is reduced. We propose that this may be due to the fact that this agency component mainly relies on the conscious metacognitive ability to recognize oneself as the author of one’s actions. Consequently, it may persist regardless of whether the avatar in the virtual environment is moving or still.

These considerations align with the predictions of the Cue Integration Theory. This theory states that the sense of agency is a multidimensional phenomenon that arises from the weighted integration of sensorimotor, proprioceptive, and exteroceptive cues and cognitive cues. In this context, the implicit dimension of agency relies more on sensorimotor signals and predictive processes, while cognitive cues, such as prior beliefs, play a more prominent role in forming the explicit judgment of agency.

## 5. Conclusions

Our findings significantly enrich our understanding of agency in VR. Indeed, they point to challenges in designing virtual environments that effectively mimic the sensorimotor feedback necessary for the arising of the implicit experience of agency. Specifically, our results suggest that what matters is not necessarily continuous online feedback of one’s actions during task execution, but rather the establishment of a coherent and reliable sensorimotor mapping between the user’s movements and the virtual body and environment. When such mapping was less coherent (e.g., in the "still avatar" condition), evidence of implicit agency was weaker. Conversely, implicit agency was observed in the experiment involving stable visuomotor correspondence during familiarisation with a responsive avatar, even though attention during the task was directed exclusively to the action-outcome. More generally, this aligns with theoretical accounts linking agency to the consistency between intentions, motor predictions, and sensory consequences [66,67], suggesting that VR designs should prioritise sensorimotor reliability and embodied plausibility to support users’ implicit sense that actions unfold as expected.

This study presents some main limitations, which are closely related. First, the two experiments were conducted on two separate and independent groups of participants. We choose this design, to avoid potential confounding effects. Specifically, we were concerned that exposure to the embodiment procedure could influence responses in the no-embodiment condition, even if the order of presentation was counterbalanced. This could have led to carryover or learning effects. However, as a result, the findings from Experiment 1 (“still avatar”) and Experiment 2 (“responsive avatar”) are not directly comparable. We can only conclude that the implicit sense of agency was not observed when participants were presented with a still-avatar, whereas it was present in the case of a responsive-avatar. Moreover, Experiment 2 differed from Experiment 1 not only in avatar responsiveness, but also in other aspects of the procedure, including the presence of a mirror during the familiarisation phase and a different response interface. Therefore, while we interpret our findings in terms of enhanced visuomotor congruency, we cannot exclude that these additional factors may have contributed to the observed differences. The implicit-agency findings from Experiment 2 should also be interpreted cautiously, given that the predefined outlier exclusion procedure resulted in the exclusion of approximately 11% of participants from this analysis.

Second, the explicit sense of ownership was assessed only in Experiment 2, and not in Experiment 1. Consequently, the study lacks a proper comparison between an experimental condition (i.e., a condition with embodiment) and a true control condition (i.e., a condition without embodiment) within the same sample. Third, during the main task, participants were instructed to focus on the lightbulb (the action-outcome), and the hand/button and the outcome could not be viewed simultaneously. While this choice was deliberate to ensure consistent processing of the action consequence, it prevents us from testing how the simultaneous availability of action and outcome cues in VR may further change the agency experience. Future research may address this limitation by developing setups in which action execution and its virtual consequence can be concurrently visible, thereby enabling a fully closed-loop action–outcome coupling. Ideally, these setups should also include integrated eye-tracking to objectively monitor gaze behaviour and attentional allocation throughout the task. A further limitation concerns avatar appearance in Experiment 2 (“responsive avatar”). Participants were assigned a gender-matched avatar, but other visually salient features (e.g., skin tone and more fine-grained appearance characteristics) were not systematically matched or individualised. Gender matching was prioritised as a pragmatic control to reduce gross incongruence in body representation, while the present study was not designed to test which specific visual traits drive ownership in VR. However, mismatches in appearance could have influenced the strength of embodiment and ownership. Future research should address this issue by using more individualised avatars and systematically manipulating appearance-related features to quantify their contribution to ownership and agency measures.

Finally, investigating how sense of agency and sense of ownership interact and at what levels (e.g., implicit vs. explicit) is a promising area for further study in VR. Indeed, VR offers unique opportunities to explore these dimensions in ways that may not be feasible in traditional experimental settings. For example, VR can allow the manipulation of agency while disrupting ownership, such as when participants control a wooden board through their own movements [68]. This potential for fine-tuned modulation makes VR a valuable tool for investigating the complexities of the sense of agency and ownership.

## References

[1] Girondini M, Mariano M, Stanco G, Gallace A, Zapparoli L. Human bodies in virtual worlds: a systematic review of implicit sense of agency and ownership measured in immersive virtual reality environments. Front Hum Neurosci. 2025;19:1553574. doi: 10.3389/fnhum.2025.1553574 40852503 PMC12367803

[2] Konstantina Kilteni, Raphaela Groten, and Mel Slater. 2012. The sense of embodiment in virtual reality. Presence: Teleoper. Virtual Environ. 21, 4 (December 2012), 373–387. 10.1162/PRES_a_00124

[3] Tieri G, Morone G, Paolucci S, Iosa M. Virtual reality in cognitive and motor rehabilitation: facts, fiction and fallacies. Expert Rev Med Devices. 2018;15(2):107–17. doi: 10.1080/17434440.2018.1425613 29313388

[4] Piccione J, Collett J, De Foe A. Virtual skills training: the role of presence and agency. Heliyon. 2019;5(11):e02583. doi: 10.1016/j.heliyon.2019.e02583 31840112 PMC6893068

[5] Riva G, Castelnuovo G, Mantovani F. Transformation of flow in rehabilitation: the role of advanced communication technologies. Behav Res Methods. 2006;38(2):237–44. doi: 10.3758/bf03192775 16956100

[6] Tsakiris M, Carpenter L, James D, Fotopoulou A. Hands only illusion: multisensory integration elicits sense of ownership for body parts but not for non-corporeal objects. Exp Brain Res. 2010;204(3):343–52. doi: 10.1007/s00221-009-2039-3 19820918

[7] Kalckert A, Ehrsson HH. Moving a rubber hand that feels like your own: a dissociation of ownership and agency. Front Hum Neurosci. 2012;6:40. doi: 10.3389/fnhum.2012.00040 22435056 PMC3303087

[8] Synofzik M, Vosgerau G, Newen A. Beyond the comparator model: a multifactorial two-step account of agency. Conscious Cogn. 2008;17(1):219–39. 17482480 10.1016/j.concog.2007.03.010

[9] Slater M, Perez-Marcos D, Ehrsson HH, Sanchez-Vives MV. Inducing illusory ownership of a virtual body. Front Neurosci. 2009;3(2):214–20. doi: 10.3389/neuro.01.029.2009 20011144 PMC2751618

[10] Haggard P. Sense of agency in the human brain. Nat Rev Neurosci. 2017;18(4):196–207. doi: 10.1038/nrn.2017.14 28251993

[11] Synofzik M, Vosgerau G, Newen A. I move, therefore I am: a new theoretical framework to investigate agency and ownership. Conscious Cogn. 2008;17(2):411–24. 18411059 10.1016/j.concog.2008.03.008

[12] Moore JW, Wegner DM, Haggard P. Modulating the sense of agency with external cues. Conscious Cogn. 2009;18(4):1056–64. doi: 10.1016/j.concog.2009.05.004 19515577

[13] Frith CD, Blakemore SJ, Wolpert DM. Abnormalities in the awareness and control of action. Philos Trans R Soc Lond B Biol Sci. 2000;355(1404):1771–88. doi: 10.1098/rstb.2000.0734 11205340 PMC1692910

[14] Wegner DM, Wheatley T. Apparent mental causation. Sources of the experience of will. Am Psychol. 1999;54(7):480–92. doi: 10.1037//0003-066x.54.7.480 10424155

[15] Wegner DM. The mind’s best trick: how we experience conscious will. Trends Cogn Sci. 2003;7(2):65–9. doi: 10.1016/s1364-6613(03)00002-0 12584024

[16] Metcalfe J, Eich TS, Castel AD. Metacognition of agency across the lifespan. Cognition. 2010;116(2):267–82. 20570251 10.1016/j.cognition.2010.05.009

[17] Haggard P, Clark S, Kalogeras J. Voluntary action and conscious awareness. Nat Neurosci. 2002;5(4):382–5. doi: 10.1038/nn827 11896397

[18] Haggard P, et al. Awareness of action in schizophrenia. Neuroreport. 2003;14(7):1081–5. 12802207 10.1097/01.wnr.0000073684.00308.c0

[19] Moore JW, Obhi SS. Intentional binding and the sense of agency: a review. Conscious Cogn. 2012;21(1):546–61. doi: 10.1016/j.concog.2011.12.002 22240158

[20] Cravo AM, Claessens PME, Baldo MVC. Voluntary action and causality in temporal binding. Exp Brain Res. 2009;199(1):95–9. doi: 10.1007/s00221-009-1969-0 19680639

[21] Zapparoli L, Seghezzi S, Zirone E, Guidali G, Tettamanti M, Banfi G, et al. How the effects of actions become our own. Sci Adv. 2020;6(27):eaay8301. doi: 10.1126/sciadv.aay8301 32937445 PMC7458439

[22] Zapparoli L, Seghezzi S, Devoto F, Mariano M, Banfi G, Porta M, et al. Altered sense of agency in Gilles de la Tourette syndrome: behavioural, clinical and functional magnetic resonance imaging findings. Brain Commun. 2020;2(2):fcaa204. doi: 10.1093/braincomms/fcaa204 33409491 PMC7772095

[23] Voss M, Moore J, Hauser M, Gallinat J, Heinz A, Haggard P. Altered awareness of action in schizophrenia: a specific deficit in predicting action consequences. Brain. 2010;133(10):3104–12. doi: 10.1093/brain/awq152 20685805

[24] Seghezzi S, Convertino L, Zapparoli L. Sense of agency disturbances in movement disorders: a comprehensive review. Conscious Cogn. 2021;96:103228. doi: 10.1016/j.concog.2021.103228 34715456

[25] Rose T, Nam CS, Chen KB. Immersion of virtual reality for rehabilitation - Review. Appl Ergon. 2018;69:153–61. doi: 10.1016/j.apergo.2018.01.009 29477323

[26] Mirelman A, Maidan I, Herman T, Deutsch JE, Giladi N, Hausdorff JM. Virtual reality for gait training: can it induce motor learning to enhance complex walking and reduce fall risk in patients with Parkinson’s disease? J Gerontol A Biol Sci Med Sci. 2011;66(2):234–40. doi: 10.1093/gerona/glq201 21106702

[27] Saposnik G, Teasell R, Mamdani M, Hall J, McIlroy W, Cheung D, et al. Effectiveness of virtual reality using Wii gaming technology in stroke rehabilitation: a pilot randomized clinical trial and proof of principle. Stroke. 2010;41(7):1477–84. doi: 10.1161/STROKEAHA.110.584979 20508185 PMC4879973

[28] Jack D, Boian R, Merians AS, Tremaine M, Burdea GC, Adamovich SV, et al. Virtual reality-enhanced stroke rehabilitation. IEEE Trans Neural Syst Rehabil Eng. 2001;9(3):308–18. doi: 10.1109/7333.948460 11561668

[29] Osumi M, Ichinose A, Sumitani M, Wake N, Sano Y, Yozu A, et al. Restoring movement representation and alleviating phantom limb pain through short-term neurorehabilitation with a virtual reality system. Eur J Pain. 2017;21(1):140–7. doi: 10.1002/ejp.910 27378656

[30] Murray CD, et al. Can immersive virtual reality reduce phantom limb pain? Stud Health Technol Inform. 2006;119:407–12. 16404088

[31] Pavão SL, Arnoni JLB, de Oliveira AKC, Rocha NACF. Impact of a virtual reality-based intervention on motor performance and balance of a child with cerebral palsy: a case study. Rev Paul Pediatr. 2014;32(4):389–94. doi: 10.1016/j.rpped.2014.04.005 25511004 PMC4311794

[32] Dinomais M, Veaux F, Yamaguchi T, Richard P, Richard I, Nguyen S. A new virtual reality tool for unilateral cerebral palsy rehabilitation: two single-case studies. Dev Neurorehabil. 2013;16(6):418–22. doi: 10.3109/17518423.2013.778347 23845037

[33] Mariano M, Stanco G, Graps DI, Rossetti I, Bolognini N, Paulesu E, et al. The sense of agency in near and far space. Conscious Cogn. 2024;120:103672. doi: 10.1016/j.concog.2024.103672 38452630

[34] Bourdin P, Martini M, Sanchez-Vives MV. Altered visual feedback from an embodied avatar unconsciously influences movement amplitude and muscle activity. Sci Rep. 2019;9(1):19747. doi: 10.1038/s41598-019-56034-5 31874987 PMC6930246

[35] Bovet S, Debarba HG, Herbelin B, Molla E, Boulic R. The critical role of self-contact for embodiment in virtual reality. IEEE Trans Vis Comput Graph. 2018;24(4):1428–36. doi: 10.1109/TVCG.2018.2794658 29543161

[36] Marchesotti S, Bernasconi F, Rognini G, De Lucia M, Bleuler H, Blanke O. Neural signatures of visuo-motor integration during human-robot interactions. Front Neurorobot. 2023;16:1034615. doi: 10.3389/fnbot.2022.1034615 36776553 PMC9908758

[37] Dewe H, Sill O, Thurlbeck S, Kentridge RW, Cowie D. The role of visuomotor synchrony on virtual full-body illusions in children and adults. J Neuropsychol. 2025;19 Suppl 1(Suppl 1):57–74. doi: 10.1111/jnp.12372 38721996 PMC11923731

[38] Jung M, et al. Impact of personalized avatars and motion synchrony on embodiment and users’ subjective experience: empirical study. JMIR Serious Games. 2022;10(4):e40119. 10.2196/40119 PMC9682455 36346658

[39] Kannape OA, Schwabe L, Tadi T, Blanke O. The limits of agency in walking humans. Neuropsychologia. 2010;48(6):1628–36. doi: 10.1016/j.neuropsychologia.2010.02.005 20144893

[40] Monti A, Porciello G, Tieri G, Aglioti SM. The “embreathment” illusion highlights the role of breathing in corporeal awareness. J Neurophysiol. 2020;123(1):420–7. doi: 10.1152/jn.00617.2019 31800367 PMC6985859

[41] Raoul L, Goulon C, Sarlegna F, Grosbras M-H. Developmental changes of bodily self-consciousness in adolescent girls. Sci Rep. 2024;14(1):11296. doi: 10.1038/s41598-024-61253-6 38760391 PMC11101456

[42] Steptoe W, Steed A, Slater M. Human tails: ownership and control of extended humanoid avatars. IEEE Trans Vis Comput Graph. 2013;19(4):583–90. doi: 10.1109/TVCG.2013.32 23428442

[43] Wiesing M, Zimmermann E. Intentional binding - Is it just causal binding? A replication study of Suzuki *et al*. (2019). Conscious Cogn. 2024;119:103665. doi: 10.1016/j.concog.2024.103665 38354485

[44] Kong G, He K, Wei K. Sensorimotor experience in virtual reality enhances sense of agency associated with an avatar. Conscious Cogn. 2017;52:115–24. doi: 10.1016/j.concog.2017.04.018 28501698

[45] Nataraj R, Sanford S, Shah A, Liu M. Agency and performance of reach-to-grasp with modified control of a virtual hand: implications for rehabilitation. Front Hum Neurosci. 2020;14:126. doi: 10.3389/fnhum.2020.00126 32390812 PMC7191072

[46] Zopf R, Polito V, Moore J. Revisiting the link between body and agency: visual movement congruency enhances intentional binding but is not body-specific. Sci Rep. 2018;8(1):196. doi: 10.1038/s41598-017-18492-7 29317726 PMC5760573

[47] Sun Y, Zhu R, Hommel B, Ma K. Social exclusion in a virtual Cyberball game reduces the virtual hand illusion. Psychon Bull Rev. 2024;31(5):2345–56. doi: 10.3758/s13423-024-02456-w 38565842

[48] Ma K, Qu J, Yang L, Zhao W, Hommel B. Explicit and implicit measures of body ownership and agency: affected by the same manipulations and yet independent. Exp Brain Res. 2021;239(7):2159–70. doi: 10.1007/s00221-021-06125-5 33974114

[49] Qu J, Ma K, Hommel B. Cognitive load dissociates explicit and implicit measures of body ownership and agency. Psychon Bull Rev. 2021;28(5):1567–78. doi: 10.3758/s13423-021-01931-y 34033062

[50] Suzuki K, et al. Intentional binding without intentional action. Psychol Sci. 2019;30(6):842–53. 31023161 10.1177/0956797619842191

[51] Nataraj R, et al. Disproportionate positive feedback facilitates sense of agency and performance for a reaching movement task with a virtual hand. PLoS One. 2020;15(5):e0233175. 10.1371/journal.pone.0233175 PMC7239468 32433665

[52] Cesari V, D’Aversa S, Piarulli A, Melfi F, Gemignani A, Menicucci D. Sense of agency and skills learning in virtual-mediated environment: a systematic review. Brain Sci. 2024;14(4):350. doi: 10.3390/brainsci14040350 38672002 PMC11048251

[53] Kong G, Aberkane C, Desoche C, Farnè A, Vernet M. No evidence in favor of the existence of “intentional” binding. J Exp Psychol Hum Percept Perform. 2024 Jun;50(6):626-635. 10.1037/xhp0001204. Epub 2024 Apr 18. 38635224

[54] Evangelou G, Georgiou O, Moore J. Using virtual objects with hand-tracking: the effects of visual congruence and mid-air haptics on sense of agency. IEEE Trans Haptics. 2023;16(4):580–5. doi: 10.1109/TOH.2023.3274304 37155385

[55] Seghezzi S, Zapparoli L. Predicting the sensory consequences of self-generated actions: pre-supplementary motor area as supra-modal hub in the sense of agency experience. Brain Sci. 2020;10(11):825. doi: 10.3390/brainsci10110825 33171715 PMC7694977

[56] Mariano M, Kuster N, Tartufoli M, Zapparoli L. How aging shapes our sense of agency. Psychon Bull Rev. 2024;31(4):1714–22. doi: 10.3758/s13423-023-02449-1 38243031 PMC11358310

[57] Jamovi. jamovi (Version 2.4) [Computer Software]; 2023.

[58] Wen W. Does delay in feedback diminish sense of agency? A review. Conscious Cogn. 2019;73:102759. doi: 10.1016/j.concog.2019.05.007 31173998

[59] Wilder TJ. Exploratory data analysis; 1977.

[60] Wobbrock JO, et al. The aligned rank transform for nonparametric factorial analyses using only ANOVA procedures. 2011.

[61] González-Franco, et al. The contribution of real-time mirror reflections of motor actions on virtual body ownership in an immersive virtual environment. 2010.

[62] Rossetti I, et al. Defective embodiment of alien hand uncovers altered sensorimotor integration in schizophrenia. Schizophr Bull. 2020;46(2):294–302. 31150551 10.1093/schbul/sbz050 PMC7406197

[63] Sivak M, Flannagan MJ, Sato T, Traube EC, Aoki M. Reaction times to neon, LED, and fast incandescent brake lamps. Ergonomics. 1994;37(6):989–94. doi: 10.1080/00140139408963712 8026456

[64] Zapparoli L, Mariano M, Sacheli LM, Berni T, Negrone C, Toneatto C, et al. Self-other distinction modulates the sense of self-agency during joint actions. Sci Rep. 2024;14(1):30055. doi: 10.1038/s41598-024-80880-7 39627377 PMC11615402

[65] Zapparoli L, Paulesu E, Mariano M, Ravani A, Sacheli LM. The sense of agency in joint actions: a theory-driven meta-analysis. Cortex. 2022;148:99–120. doi: 10.1016/j.cortex.2022.01.002 35168155

[66] Evans N, Gale S, Schurger A, Blanke O. Visual feedback dominates the sense of agency for brain-machine actions. PLoS One. 2015;10(6):e0130019. doi: 10.1371/journal.pone.0130019 26066840 PMC4466540

[67] Tsakiris M, Haggard P, Franck N, Mainy N, Sirigu A. A specific role for efferent information in self-recognition. Cognition. 2005;96(3):215–31. doi: 10.1016/j.cognition.2004.08.002 15996559

[68] Pyasik M, Tieri G, Pia L. Visual appearance of the virtual hand affects embodiment in the virtual hand illusion. Sci Rep. 2020;10(1):5412. doi: 10.1038/s41598-020-62394-0 32214171 PMC7096421

